# Evaluation of SARS-CoV-2 passive surveillance in Lithuanian mink farms, 2020–2021

**DOI:** 10.3389/fvets.2023.1181826

**Published:** 2023-06-09

**Authors:** Silvija Žigaitė, Marius Masiulis, Paulius Bušauskas, Simona Pilevičienė, Jūratė Buitkuvienė, Vidmantas Paulauskas, Alvydas Malakauskas

**Affiliations:** ^1^Veterinary Academy, Lithuanian University of Health Sciences, Kaunas, Lithuania; ^2^State Food and Veterinary Service, Vilnius, Lithuania; ^3^National Food and Veterinary Risk Assessment Institute, Vilnius, Lithuania

**Keywords:** SARS-CoV-2, COVID-19, mink, *Neovison vison*, surveillance, Lithuania

## Abstract

The newly emerged SARS-CoV-2, causing COVID-19 in humans, is also infecting American mink (*Neovison vison*), used in fur production. Since 2020, passive surveillance of SARS-CoV-2 in mink farms was implemented in Lithuania. Here, we describe data from a survey of all 57 active Lithuanian mink farms carried out during November–December 2021 to complement passive surveillance in the country. In all 57 mink farms, nasopharyngeal swab samples were collected from dead or live mink and tested by real-time RT-PCR. Dead mink samples were tested in pools of 5, while live mink samples were tested individually. In 19 mink farms, blood serum was collected and tested for antibodies to determine previous exposure to the virus. Environmental samples from 55 farms were also collected and tested in pooled samples by real-time RT-PCR. The present survey has detected 22.81% viral RNA-positive mink farms and a high number of mink farms that were exposed (84.21, 95% CI 67.81–100%) to the virus. The increasing exposure of mink farms to the virus due to growing human COVID-19 cases and limitations of passive surveillance could explain the observed epidemiological situation of SARS-CoV-2 in Lithuanian mink farms, compared to the few positive farms previously detected by passive surveillance. The unexpected widespread exposure of mink farms to SARS-CoV-2 suggests that passive surveillance is ineffective for early detection of SARS-CoV-2 in mink. Further studies are needed to reveal the present status in previously infected mink farms.

## 1. Introduction

The newly emerged severe acute respiratory syndrome coronavirus 2 (SARS-CoV-2), causing coronavirus disease 19 (COVID-19), was first detected in humans in December 2019 and soon became a global pandemic (1). Susceptibility to the virus was confirmed in various mammal species as a result of contact with infected humans (2). By July 2022, 35 countries have reported the infection in 24 different animal species to the World Organisation for Animal Health (3). Among these species, American mink (*Neovison vison*), used in fur production, was found to be especially susceptible to SARS-CoV-2 infection. After the first report in Dutch mink farms in April 2020 (4), the virus was reported in 12 more countries—Canada, Denmark, France, Greece, Italy, Latvia, Lithuania, Netherlands, Poland, Spain, Sweden, and USA (5). Furthermore, a few reports indicate that feral and escaped minks have also been infected (6, 7), and mink-associated SARS-CoV-2 spill-over to humans has been observed in the Netherlands, Denmark, Poland, and possibly the USA (8, 9). The virus spreads effectively through mink and thus accumulates mutations. Mink-associated virus variants with amino acid changes in the spike protein demonstrated reduced sensitivity to neutralizing antibodies (10). The risk of infection with mink-related virus strains is highest for mink farm workers (2). There is also a risk for other farm animals since cats and dogs were found infected under field conditions (11). Furthermore, there is a risk of establishing a SARS-CoV-2 reservoir in areas with a high density of mink farms or stable wild mink populations (9).

According to European Food Safety Authority (EFSA) and European Centre for Disease Prevention and Control (ECDC), all mink farms are at risk of infection and should be under surveillance (12). An active monitoring approach with the main objective of the early detection of the virus has been recommended by EFSA (2). Active surveillance is highly resource-demanding. Therefore, alternative passive surveillance and a risk-based approach could be implemented (13). Passive surveillance has the potential of under-reporting due to various factors, including farmers’ overall disease awareness and decision-making (14).

Since 2020, the State Food and Veterinary Service of the Republic of Lithuania (SFVS) implemented mandatory passive surveillance of the virus in the country’s mink farms. Mink farm owners were obligated to report higher than usual mink mortality/morbidity rates, reduced feed consumption, and confirmed COVID-19 infection in farm personnel to territorial SFVS. Furthermore, mink farms had to provide factual numbers of mink mortality and morbidity to territorial SFVS on a weekly basis. In November 2020, the SFVS carried out a sampling of dead minks in all active mink farms in the country with negative results, although relatively few nasopharyngeal swab samples were tested per farm by real-time RT-PCR (89 samples from 69 mink farms). In November and December 2020, the first two SARS-CoV-2 infected mink farms have been detected by passive surveillance in Lithuania (15). While numerous outbreaks were detected in Europe from the start of 2021, only two more mink farms were found infected through passive surveillance in Lithuania at the beginning of 2021 (15). Furthermore, in October and November 2021, SARS-CoV-2 spill-overs from mink to humans were identified by Lithuania’s SARS-CoV-2 genomic surveillance program (unpublished data, reported by G. Dudas). Considering the potential of under-reporting in passive surveillance and increasing numbers of human COVID-19 cases since September 2021, a survey was performed to investigate SARS-CoV-2 in mink farms in Lithuania. We report the survey results of the SARS-CoV-2 infection and exposure in Lithuanian mink farms, performed in November–December 2021.

## 2. Materials and methods

In November–December 2021, according to an order of SFVS, all active mink farms (i.e., live minks present on the farm) in Lithuania had to be sampled and tested for SARS-CoV-2. Sampling on farms was done by official veterinarians. During the survey, no movement of minks was allowed in the country.

At least 30 nasopharyngeal swab samples from either dead, sick or live mink were taken at every mink farm, with the aim of detecting a 10% within-farm infection prevalence at 95% confidence. In very few cases, less than 30 samples were taken due to intensive pelting and high workload, as well as in some farms—more than 30 samples were taken, where more than one epidemiological unit was present. The priority was to take samples from dead and diseased minks. The rest of the samples were taken from minks killed for pelting or live animals. In the latter situation, samples were collected to ensure that each sector of a farm was sampled.

Nineteen mink farms out of 57 were convenience sampled for mink blood serum to determine previous exposure to the virus. Thirty mink blood samples were collected from each farm. Both adult (more than 1-year-old) and juvenile (less than 1-year-old) minks were sampled from various places of a farm. Additionally, environmental swab samples were collected from 55 mink farms using the same swabs that were used for nasopharyngeal sample collection. Each farm was divided by area size into five roughly equal parts, and one sample was taken from every area. Environmental swab samples were collected from surfaces of mink cages, walls, ceilings, and floors of open houses, as well as from household items that had contact with the minks or farm staff. Risk-based sampling was performed as samples were first collected from open houses with increased mink mortality or morbidity if present.

Information about the exact location of every taken mink or environmental sample was collected, as well as the total number of minks present on farms. Information about the age of sampled minks was collected from most of the farms. All samples from one farm were collected and delivered to the laboratory on the same day. All laboratory testing was done at the National Food and Veterinary Risk Assessment Institute in Lithuania.

### 2.1. Real-time RT-PCR testing

Swab samples from dead mink and the environment were tested by real-time reverse transcription-polymerase chain reaction (real-time RT-PCR) in pools of 5 individual samples from the same mink farm. Live mink swab samples were tested individually. All samples were kept so they could be tested individually. MagMAX^™^ Viral/Pathogen II (MVP II) Nucleic Acid Isolation Kit (Applied Biosystems, Thermo Fisher Scientific) and KingFisher Flex system (Thermo Fisher Scientific) were used for viral RNA extraction, and TaqPath^™^ COVID-19 RT-PCR Kit (Applied Biosystems, Thermo Fisher Scientific) was used for real-time RT-PCR reaction, according to the manufacturer’s protocol.

### 2.2. Enzyme-linked immunosorbent assay (ELISA) testing

Mink blood serum samples were tested individually. Blood samples were centrifuged, and the serum was collected. ID Screen SARS-CoV-2 Double Antigen Multi-Species kit (ID.VET) was used to detect anti-SARS-CoV-2 antibodies in mink serum. The solutions were prepared, and testing was done according to the manufacturer’s protocol.

### 2.3. Statistical analysis

Microsoft Excel Spreadsheet Software (Microsoft Office Standard 2019, version 1808) and Epitools software were used for statistical analysis. A 2-sample *z*-test for sample proportion comparison^1^ was used to compare PCR-positive and antibody-positive farms, and to calculate confidence intervals. A Chi-squared test^2^ was used to compare positive and negative samples taken from adult and juvenile minks.

### 2.4. Ethics statement

No experimental procedures were performed on animals. All animal samples were taken by official veterinarians for a compulsory animal health surveillance program. Therefore, no ethical approval was required.

## 3. Results

During the survey period, a total of 57 mink farms in Lithuania were tested for SARS-CoV-2, and 25 were found positive (43.86, 95% CI 30.98–56.74%) either by RT-PCR for the presence of the virus or ELISA for the presence of antibodies. None of the sampled farms reported increased mortality or morbidity during the study. The positive farms were situated across the country with no signs of obvious clustering. At the time of sampling, the number of mink present on all sampled farms varied widely and ranged from 120 to 159,916, with an average of 14,597 animals present on farms (Supplementary Table S1).

Information about infected staff in mink farms showed that in 11 farms, at least one staff was confirmed to have been infected with SARS-CoV-2 from 4 days up to 1 year before a farm was sampled. Four of these farms were viral RNA-positive, and 10 of them were positive for anti-SARS-CoV-2 antibodies. No infected staff was reported in SARS-CoV-2-negative farms (Supplementary Table S1).

### 3.1. RT-PCR testing of mink nasopharyngeal swabs

In total, 13 (22.81%) out of 57 tested farms were found SARS-CoV-2 RT-PCR positive (Table 1).

**Table 1:** Real-time RT-PCR test results of SARS-CoV-2 survey in mink farms in Lithuania in November–December 2021.

	No. of farms tested	Average no. of mink present on negative farms	Average no. of mink present on positive farms	No. of mink tested	No. of pools tested*	Positive samples**		Positive farms	
						No.	% (95% CI)	No.	% (95% CI)
Swab samples collected from only dead mink	22	24,702	10,956	647	134	21	15.67 (9.52–21.82)	6	27.27 (8.66–45.88)
Swab samples collected from only live mink	23	7,681	7,807	670	n.d.	15	2.24 (1.12–3.36)	3	13.04 (0.0–26.8)
Swab samples collected from dead and live mink	12	18,290	11,918	641 (296 dead +345 live)	60	16 (8 pools +8 individual)	3.95 (2.05–5.85)	4	33.33 (6.66–60)
Total swab samples	57	15,800	10,525	1,958	194	52	4.3 (3.16–5.44)	13	22.81

*Dead mink nasopharyngeal swab samples tested in pools of 5 by real-time RT-PCR.

**Positive samples are presented as pooled positive samples for tested dead mink, as individual positive samples for tested live mink, and as a combination of pooled and individual positive samples where both dead and live mink were tested.

n.d., swab samples from live mink were tested individually, no pooling was done.

A total of 943 dead mink nasopharyngeal swab samples were taken from 34 mink farms. Data about the age of tested dead mink were collected from 26 farms. Seventeen pooled samples were taken and tested from adult dead mink, and one pooled sample (5.88, 95% CI 0.0–17.06%) tested positive for viral RNA. Meanwhile, 140 pooled samples from juvenile mink were tested, and 19 of them (13.57, 95% CI 7.9–19.24%) tested positive by real-time RT-PCR (Supplementary Table S2). There was no statistically significant difference between the proportions of viral RNA-positive samples taken from adult versus juvenile dead minks (*p* = 0.6081).

In total, 1,015 live mink swab samples were collected from 35 farms. Data about the age of tested live mink were collected from 22 of these farms. Samples from 76 adult live mink were collected, and 4 of them (5.26, 95% CI 0.24–10.28%) tested positive for viral RNA. Meanwhile, 565 juvenile live mink samples were collected, and 15 of them (2.65, 95% CI 1.33–3.97%) tested positive by real-time RT-PCR (Supplementary Table S3). The difference between viral RNA-positive and negative samples taken from adult versus juvenile live minks was not statistically significant (*p* = 0.3689).

### 3.2. RT-PCR testing of environmental samples

A total of 55 pooled environmental swab samples were collected from 55 different mink farms, five samples each. From one farm out of 55 (1.82, 95% CI 0.0–5.35%), only one of the five individual environmental swab samples tested positive for SARS-CoV-2 RNA. The positive sample was taken from the surface of a mink cage. Dead and live mink nasopharyngeal swab samples collected from this farm also tested positive for viral RNA (Supplementary Table S1), but no blood serum samples were available for testing.

### 3.3. Mink serum ELISA testing

**Figure 1:**
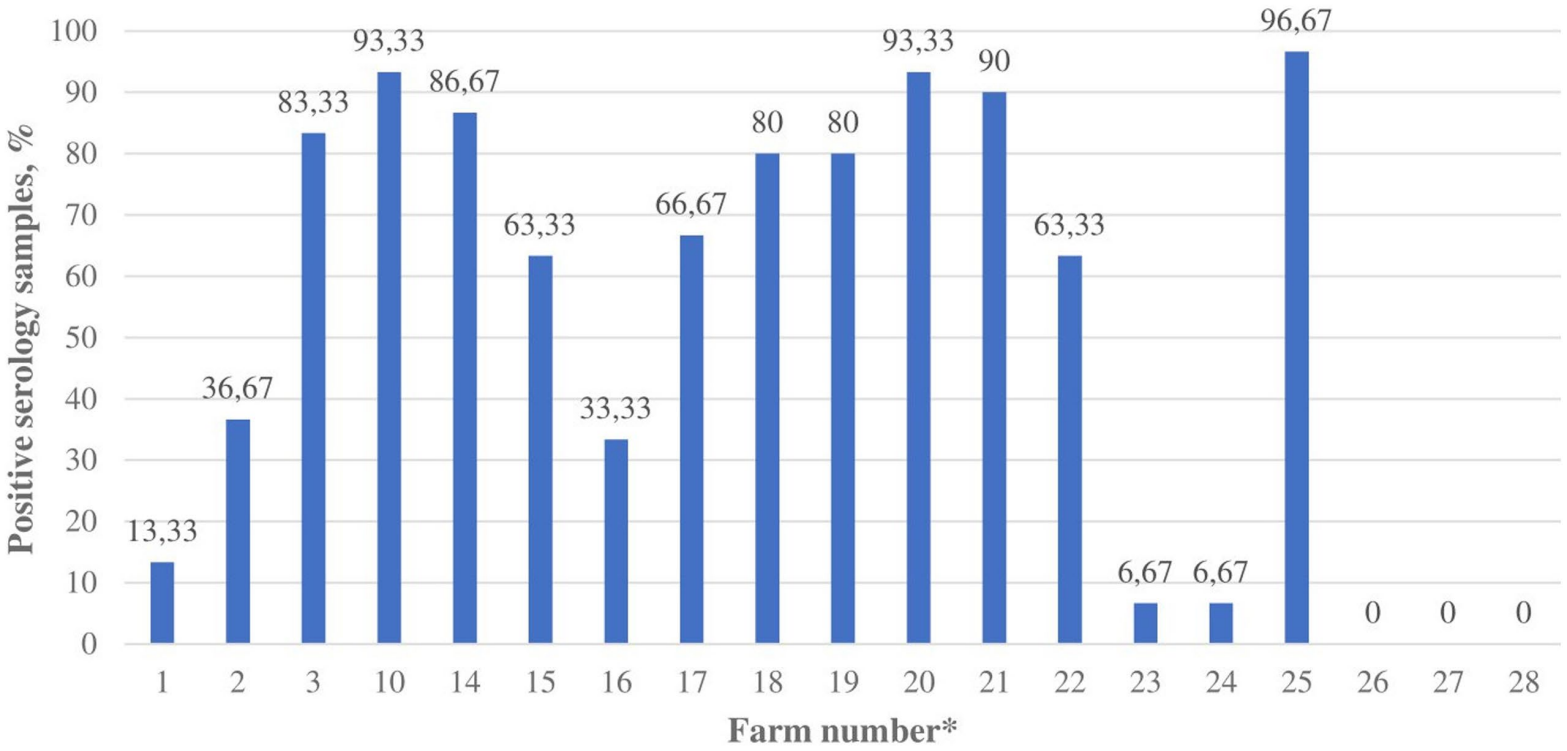
The proportion of blood serum samples with anti-SARS-CoV-2 antibodies in Lithuanian mink farms (*n* = 19), November–December 2021. * Farm number corresponds to the farm numbers in Supplementary Table S1.

Nineteen mink farms were sampled and tested for anti-SARS-CoV-2 antibodies. The limited blood serum sampling was due to intensive swab sampling and reduced resources of veterinary and farm personnel because of the COVID-19 pandemic. In total, 570 mink serum samples from 19 farms were tested by ELISA. Out of these, 298 mink samples from 16 (84.21, 95% CI 67.81–100%) farms tested positive for anti-SARS-CoV-2 antibodies (Figure 1). Only 4 of the antibody-positive farms were also viral RNA-positive.

At the time of sampling, the number of minks on the 19 serologically tested farms ranged from 1,025 to 79,300. The average number of minks on the antibody-positive farms was 14,992 and 7,298 on the antibody-negative farms (Supplementary Table S1). Data about the age of sampled mink were collected from 13 farms. Serum samples from 79 adult minks were collected, and 29 of them (36.71, 95% CI 26.08–47.34%) tested positive for anti-SARS-CoV-2 antibodies. Serum samples were collected and tested from 311 juvenile minks, and 136 of them (43.73, 95% CI 38.22–49.24%) tested positive by ELISA (Supplementary Table S4). The difference between antibody-positive and negative samples taken from adult versus juvenile minks was not statistically significant (*p* = 0.3171).

The proportion of mink farms with anti-SARS-CoV-2 antibodies was significantly (*p* < 0.0001) higher than the proportion of viral RNA-positive farms.

## 4. Discussion

This study was carried out to complement the ongoing passive surveillance of SARS-CoV-2 in the Lithuanian mink farm population during the COVID-19 pandemic and continuous reports of mink SARS-CoV-2 infection from various countries. The present survey, implemented in November–December 2021, has revealed considerably more SARS-CoV-2 RNA-positive mink farms (13 farms, Table 1) than was detected (4 farms) by passive surveillance in November 2020–October 2021. This survey also revealed an unexpected widespread exposure of mink farms (84.21, 95% CI 67.81–100%) to the virus, which is evident by the presence of anti-SARS-CoV-2 antibodies in mink.

The detected epidemiological situation is the result of increasing exposure of mink farms to the virus from accumulating human COVID-19 cases in Lithuania during the second half of 2021 and limitations of passive surveillance. It has been shown in Denmark that the epidemic curve of SARS-CoV-2 in mink farms closely follows the epidemic curve of COVID-19 human cases (16). The rise of human COVID-19 cases in Lithuania has been observed in October–December 2020 and August–November 2021 (17), just before the first SARS-CoV-2 mink farm was detected by passive surveillance at the end of 2020, and before the present survey when considerably underestimated SARS-CoV-2 presence in mink farms at the end of 2021 was found, respectively. In Denmark, it has also been shown that in approx. 2 months, the number of infected farms could rise tenfold from 3 to 30 (2). The further concern is that the peak of human COVID-19 incidence in Lithuania occurred in January–February 2022, thus creating even more pressure for the virus to be transmitted to mink farms. This poses potentially dangerous possibilities for genetic mutations of the virus and a significant virus transmission risk between mink and humans in Lithuania.

COVID-19 clinical signs in mink are usually unspecific – increased mortality, mild respiratory symptoms, and decreased feed intake are observed most often, but subclinical infections also have been detected (2). In most cases, the introduction of the virus is suspected to be caused by infected humans. Therefore, the vital part of early detection monitoring should be strict periodic testing of farm personnel and other people in contact with the animals as humans are the most likely route of SARS-CoV-2 introduction into the farm (2). Once introduced in a fur farm, the virus spreads efficiently due to minks living in densely packed open houses. The contiguous cages allow for direct animal contact. SARS-CoV-2 is transmitted by direct and indirect contact (air droplets, dust particles, aerosols, and fomites). Complex biosecurity measures should be implemented on the farm to prevent the entry of the virus (2). The risk of transmission of the virus between mink and humans in Lithuania could be reduced by implementing very strict within-farm biosecurity measures (e.g., FFP respirators, goggles, hygiene, etc.), but this would be difficult to maintain at a constantly effective level for a prolonged duration as it depends on the attitude and perceptions of farm workers.

There is little data on the occurrence of SARS-CoV-2 in mink farms before the start of passive surveillance in November 2020 in Lithuania. A survey performed at the start of November 2020 has not revealed any of the active 68 farms in the country to be positive for SARS-CoV-2 infection, although only one to three nasopharyngeal swab samples were collected from dead minks. Therefore, a small sample size creates a low sensitivity of this surveillance (15). Soon after the passive surveillance started in November 2020, the first confirmed occurrence of SARS-CoV-2 in a Lithuanian mink farm has been detected. However, only this first infected mink farm was detected by passive surveillance due to the reported increased daily mortality of minks by 0.36% to SFVS. This is a little bit lower mortality than was observed (0.45%) in Denmark (16). The other infected mink farms were detected after COVID-19-infected farm workers were reported to SFVS. This information was provided from two sources – mink farmers were obligated to report to SFVS, and this information was also obtained from the National Public Health Center under the Ministry of Health, which is the official responsible authority for handling information about human COVID-19 cases.

It is not known if SARS-CoV-2 really did not cause a noticeably increased mortality and/or morbidity in Lithuanian mink farms or if it was simply not reported by the farmers, even if it was compulsory to provide the data on the mortality in mink farms on a weekly basis. The virus is known to induce subclinical infection in mink, and it has been reported in several countries like Denmark, Netherlands, France, Italy, and Greece, but clinical signs were still noticed and reported in approx. 30–42% of infected farms (2). It should be noted that clinical signs were not observed by official veterinary inspectors during the sampling in this survey. However, it could not be excluded that there was a lack of cooperation between farmers and veterinary authorities, and farmers were not willing to share information about sick animals and risk their profit and livelihood. It is also important that the passive surveillance program of SARS-CoV-2 in Lithuanian mink farms did not include any incentives that would encourage the reporting by the farmers. Meanwhile, the infected farms would have to deal with animal movement restrictions, stricter biosecurity measures, and more frequent reporting of dead and sick animals.

Underreporting is a known limitation of passive surveillance (18), and it could explain the underestimation of SARS-CoV-2 infected mink farms detected by passive surveillance in Lithuania. Another reason for this is the aforementioned lack of clinical signs and the absence of increased mortality in infected mink. The virus can cause a subclinical infection and go undetected by passive surveillance (2), which relies heavily on the observation of clinical signs (18). Most importantly, the current study was performed right after the highest spike of COVID-19 human cases was observed in Lithuania on November 6^th^, 2021 (over 58,000 active cases) (17). The increase of SARS-CoV-2 human cases is known to affect the virus prevalence in mink farms (16).

This investigation revealed that mink farms have been detected at various stages of SARS-CoV-2 infection. Antibodies were detected in 3.69 times more (*p* < 0.0001) mink farms than the SARS-CoV-2 RNA. SARS-CoV-2 infection was likely detected in different stages at various farms. For instance, 11 out of 16 antibody-positive mink farms tested negative by real-time RT-PCR, but most of them had a high proportion of antibody-positive samples, suggesting a long presence of SARS-CoV-2 infection in the farms. Furthermore, all pooled dead mink samples from farms No. 1 and 2 tested positive for viral RNA, but less than half of the tested mink blood samples had anti-SARS-CoV-2 antibodies, suggesting an earlier infection phase (Supplementary Table S1). Further studies are needed to explore if the virus could be eliminated from a mink farm due to the acquired immunity of minks. We found that the majority of mink farms (84.21, 95% CI 67.81–100%) could have already been exposed to the virus in Lithuania. This shows that passive surveillance has been ineffective for early detection of SARS-CoV-2 in mink. Therefore, early detection in Lithuanian mink farms might not be of major importance, but the monitoring of the virus evolution becomes the priority. Further studies are needed to reveal the present status in previously infected Lithuanian mink farms.

Research in Denmark, Poland, and Italy shows that the number of antibody-positive mink varies from 30 to 100% per farm, while viral RNA in these farms ranged from a few positive samples to 100% (16, 19–22), and we observed a similar tendency, using the same methods. Interestingly, our results show that testing of dead mink, even in the absence of reported increased mortality and morbidity, is almost two times more effective at detecting SARS-CoV-2 infected mink farms compared to only live mink sampling, although this difference is not statistically significant (Table 1). This could raise some doubts about the ability of mink farm workers to detect a disease in mink and/or the absence of proper reporting. However, the average number of animals present was greater on positive farms where only dead mink were tested compared to positive farms where only live mink were sampled. This could suggest that larger farms have more dead animals, and thus they would be more likely to do dead animal testing than smaller farms where dead minks are found less regularly. Mink age did not have an effect on the proportion of viral RNA-positive samples.

Despite the high number of viral RNA-positive or antibody-positive samples in some mink farms, only one individual sample, taken from the surface of a mink cage in one (1.82%) mink farm, tested positive by real-time RT-PCR in this study. The low number of positive environmental samples detected in our study could be due to the collection technique because swabs used in our study covered much less surface area than dust cloths used in Dutch and Greek studies (23, 24). Therefore, a method covering more surface area could have been useful to accurately evaluate the mink farm environment and compare it to the number of positive animals. Another limitation of this study was the lack of postmortem examination of dead mink. Therefore, we cannot evaluate if mink mortality could be related to SARS-CoV-2 infection. Testing a rather high number of active mink farms and restricted human resources due to the COVID-19 pandemic during the survey made it not possible to sample all farms uniformly and collect additional epidemiological information, thus limiting possibilities to investigate the course of SARS-CoV-2 in Lithuanian mink farms in more detail. Additionally, there is no data yet available about the circulating SARS-CoV-2 variants in Lithuanian mink farms, so we cannot evaluate the transmission of SARS-CoV-2 between mink farms and the human population in Lithuania in detail.

## 5. Conclusion

This study showed a lack of detection of SARS-CoV-2 in Lithuanian mink farms by passive surveillance. However, it remains unclear if it was caused by improper reporting or limited increase of mortality and/or morbidity of SARS-CoV-2 infected mink. The unexpected widespread exposure of mink farms (84.21, 95% CI 67.81–100%) to the virus suggests that passive surveillance is ineffective for early detection of SARS-CoV-2 in mink. Further studies are needed to reveal the present status in previously infected Lithuanian mink farms.

## Data availability statement

The raw data supporting the conclusions of this article will be made available by the authors, without undue reservation.

## Ethics statement

Ethical review and approval was not required for the animal study because no experimental procedures were performed on animals. All animal samples were taken by official veterinarians for a compulsory animal health surveillance program. Therefore, no ethical approval was required.

## Author contributions

MM, VP, PB, and AM contributed to conception and design of the study. SP and JB performed the laboratory analysis. SŽ performed the data analysis and wrote the first draft of the manuscript. AM wrote sections of the manuscript. All authors contributed to the article and approved the submitted version.

## Funding

The collection and testing of samples were done by implementing disease control services of Lithuanian State Food and Veterinary Services and was partly supported by the Lithuanian Ministry of Agriculture.

## Conflict of interest

The authors declare that the research was conducted in the absence of any commercial or financial relationships that could be construed as a potential conflict of interest.

## Publisher’s note

All claims expressed in this article are solely those of the authors and do not necessarily represent those of their affiliated organizations, or those of the publisher, the editors and the reviewers. Any product that may be evaluated in this article, or claim that may be made by its manufacturer, is not guaranteed or endorsed by the publisher.

## Footnotes

^1^ https://epitools.ausvet.com.au/ztesttwo

^2^ https://epitools.ausvet.com.au/chisqone
