# Supplementary material for: Evaluation of SARS-CoV-2 passive surveillance in Lithuanian mink farms, 2020–2021
Source: Front Vet Sci. 2023 Jun 9;10:1181826. doi: 10.3389/fvets.2023.1181826 (PMC10288870; doi:10.3389/fvets.2023.1181826)
Supplement: Supplementary file 1 [file Table_1.DOCX]

Supplementary Material

Evaluation of SARS-CoV-2 Passive Surveillance in Lithuanian Mink Farms, 2020–2021

Silvija Žigaitė*, Marius Masiulis, Paulius Bušauskas, Simona Pilevičienė, Jūratė Buitkuvienė, Vidmantas Paulauskas, Alvydas Malakauskas

*** Correspondence:** Silvija Žigaitė: silvija.zigaite@lsmu.lt

# Supplementary Figures and Tables

## Supplementary Tables

**Supplementary Table 1.** Summary of laboratory results from all active Lithuanian mink farms that were tested in November-December 2021 (numbers in bold highlight the SARS-CoV-2 positive results).

| **Farm No.** | **No. of mink present on a farm** | **No. of collected dead mink samples** | **No. of tested pools*** | **No. of positive pools*** | **No. of tested live mink** | **No. of positive live mink** | **No. of collected blood samples** | **No. of positive blood samples** | **No. of infected employees and confirmation date** |
| --- | --- | --- | --- | --- | --- | --- | --- | --- | --- |
| 1 | 15 110 | 30 | 6 | **6** | n.d. | n.d. | 30 | **4** | 1 employee, 4 days before sampling |
| 2 | 2 300 | 30 | 6 | **6** | n.d. | n.d. | 30 | **11** | 1 employee, 6 months before sampling |
| 3 | 19 428 | 45 | 9 | **1** | n.d. | n.d. | 30 | **25** | 1 employee, 18 days before sampling |
| 4 | 7 000 | 6 | 2 | **2** | n.d. | n.d. | n.d. | n.d. | None |
| 5 | 10 100 | 24 | 8 | **3** | n.d. | n.d. | n.d. | n.d. | None |
| 6 | 13 470 | 7 | 2 | **2** | 30 | 0 | n.d. | n.d. | None |
| 7 | 11 800 | 30 | 6 | **3** | n.d. | n.d. | n.d. | n.d. | None |
| 8** | 6 300 | 4 | 1 | **1** | 30 | **3** | n.d. | n.d. | None |
| 9 | 25 000 | 30 | 6 | **5** | 30 | **2** | n.d. | n.d. | None |
| 10 | 3 420 | n.d. | n.d. | n.d. | 30 | **1** | 30 | **28** | None |
| 11 | 5 000 | n.d. | n.d. | n.d. | 30 | **1** | n.d. | n.d. | None |
| 12 | 15 000 | n.d. | n.d. | n.d. | 30 | **13** | n.d. | n.d. | None |
| 13 | 2 900 | 30 | 6 | 0 | 30 | **3** | n.d. | n.d. | 1 employee, 10 days before sampling |
| 14 | 79 300 | 30 | 6 | 0 | 30 | 0 | 30 | **26** | 3 employees, 3 months before sampling |
| 15 | 10 512 | 30 | 6 | 0 | n.d. | n.d. | 30 | **19** | None |
| 16 | 1 025 | n.d. | n.d. | n.d. | 30 | 0 | 30 | **10** | 1 owner, 1.5 months before sampling (no contact with animals) |
| 17 | 39 068 | 32 | 7 | 0 | n.d. | n.d. | 30 | **20** | 1 employee, 8 months before sampling (no contact with animals) |
| 18 | 3 200 | 15 | 3 | 0 | 15 | 0 | 30 | **24** | None |
| 19 | 3 780 | n.d. | n.d. | n.d. | 30 | 0 | 30 | **24** | None |
| 20 | 21 350 | 30 | 6 | 0 | n.d. | n.d. | 30 | **28** | 2 employees, one a year before sampling and another 1.5 months before sampling |
| 21 | 20 720 | 30 | 6 | 0 | n.d. | n.d. | 30 | **27** | 2 employees, one a year before sampling and another 1.5 months before sampling |
| 22 | 9 000 | 30 | 6 | 0 | n.d. | n.d. | 30 | **19** | 1 employee, 3 months before sampling |
| 23 | 3 000 | 30 | 6 | 0 | n.d. | n.d. | 30 | **2** | None |
| 24 | 4 153 | 30 | 6 | 0 | n.d. | n.d. | 30 | **2** | None |
| 25 | 4 500 | 30 | 6 | 0 | n.d. | n.d. | 30 | **29** | 2 employees, 5 months before sampling |
| 26 | 6 481 | n.d. | n.d. | n.d. | 30 | 0 | 30 | 0 | None |
| 27 | 5 000 | 30 | 6 | 0 | n.d. | n.d. | 30 | 0 | None |
| 28 | 10 413 | 30 | 6 | 0 | n.d. | n.d. | 30 | 0 | None |
| 29 | 120 | 30 | 6 | 0 | n.d. | n.d. | n.d. | n.d. | None |
| 30 | 4 850 | 30 | 6 | 0 | 30 | 0 | n.d. | n.d. | None |
| 31 | 15 080 | 30 | 6 | 0 | n.d. | n.d. | n.d. | n.d. | None |
| 32 | 800 | 30 | 6 | 0 | 30 | 0 | n.d. | n.d. | None |
| 33 | 10 216 | 30 | 6 | 0 | 30 | 0 | n.d. | n.d. | None |
| 34 | 72 007 | 30 | 6 | 0 | n.d. | n.d. | n.d. | n.d. | None |
| 35 | 159 916 | 30 | 6 | 0 | n.d. | n.d. | n.d. | n.d. | None |
| 36 | 22 000 | 30 | 6 | 0 | 30 | 0 | n.d. | n.d. | None |
| 37 | 4 000 | 30 | 6 | 0 | 30 | 0 | n.d. | n.d. | None |
| 38 | 3 450 | 30 | 6 | 0 | n.d. | n.d. | n.d. | n.d. | None |
| 39 | 21 950 | 30 | 6 | 0 | 30 | 0 | n.d. | n.d. | None |
| 40 | 6 087 | n.d. | n.d. | n.d. | 30 | 0 | n.d. | n.d. | None |
| 41 | 1 650 | n.d. | n.d. | n.d. | 30 | 0 | n.d. | n.d. | None |
| 42 | 3 102 | n.d. | n.d. | n.d. | 30 | 0 | n.d. | n.d. | None |
| 43 | 2 500 | n.d. | n.d. | n.d. | 30 | 0 | n.d. | n.d. | None |
| 44 | 2 000 | n.d. | n.d. | n.d. | 30 | 0 | n.d. | n.d. | None |
| 45 | 1 200 | n.d. | n.d. | n.d. | 30 | 0 | n.d. | n.d. | None |
| 46 | 2 460 | n.d. | n.d. | n.d. | 30 | 0 | n.d. | n.d. | None |
| 47 | 26 340 | n.d. | n.d. | n.d. | 30 | 0 | n.d. | n.d. | None |
| 48 | 820 | n.d. | n.d. | n.d. | 11 | 0 | n.d. | n.d. | None |
| 49 | 24 764 | n.d. | n.d. | n.d. | 30 | 0 | n.d. | n.d. | None |
| 50 | 16 950 | 30 | 6 | 0 | n.d. | n.d. | n.d. | n.d. | None |
| 51 | 20 950 | n.d. | n.d. | n.d. | 30 | 0 | n.d. | n.d. | None |
| 52 | 5 094 | n.d. | n.d. | n.d. | 30 | 0 | n.d. | n.d. | None |
| 53 | 994 | n.d. | n.d. | n.d. | 30 | 0 | n.d. | n.d. | None |
| 54 | 10 950 | n.d. | n.d. | n.d. | 30 | 0 | n.d. | n.d. | None |
| 55 | 3 000 | n.d. | n.d. | n.d. | 29 | 0 | n.d. | n.d. | None |
| 56 | 20 879 | n.d. | n.d. | n.d. | 30 | 0 | n.d. | n.d. | None |
| 57 | 9 550 | n.d. | n.d. | n.d. | 30 | 0 | n.d. | n.d. | None |

* - dead mink swab samples tested in pools of 5.

** - only in farm No. 8 one out of 5 environmental samples tested positive by real-time RT-PCR.

n.d. - not done.

**Supplementary Table 2.** The number of adult (more than 1-year-old) and juvenile (less than 1-year-old) dead minks tested by real-time RT-PCR and found positive at sampled Lithuanian mink farms in November-December 2021.

| **Farm No.** | **No. of tested adult dead mink** | **No. of tested adult dead mink pools*** | **No. of positive adult dead mink pools*** | **No. of tested juvenile dead mink** | **No. of tested juvenile dead mink pools*** | **No. of positive juvenile dead mink pools*** |
| --- | --- | --- | --- | --- | --- | --- |
| 1 | 0 | 0 | 0 | 30 | 6 | 6 |
| 2 | 0 | 0 | 0 | 30 | 6 | 6 |
| 3 | 0 | 0 | 0 | 45 | 9 | 1 |
| 4 | n.a. | n.a. | n.a. | n.a. | n.a. | n.a. |
| 5 | 0 | 0 | 0 | 24 | 8 | 3 |
| 6 | n.a. | n.a. | n.a. | n.a. | n.a. | n.a. |
| 7 | 10 | 2 | 1 | 20 | 4 | 2 |
| 8 | 0 | 0 | 0 | 4 | 1 | 1 |
| 12 | n.a. | n.a. | n.a. | n.a. | n.a. | n.a. |
| 13 | 0 | 0 | 0 | 30 | 6 | 0 |
| 14 | 0 | 0 | 0 | 30 | 6 | 0 |
| 15 | 15 | 3 | 0 | 15 | 3 | 0 |
| 17 | 0 | 0 | 0 | 32 | 7 | 0 |
| 18 | n.a. | n.a. | n.a. | n.a. | n.a. | n.a. |
| 20 | n.a. | n.a. | n.a. | n.a. | n.a. | n.a. |
| 21 | n.a. | n.a. | n.a. | n.a. | n.a. | n.a. |
| 22 | 30 | 6 | 0 | 0 | 0 | 0 |
| 23 | 0 | 0 | 0 | 30 | 6 | 0 |
| 24 | n.a. | n.a. | n.a. | n.a. | n.a. | n.a. |
| 25 | 0 | 0 | 0 | 30 | 6 | 0 |
| 27 | 0 | 0 | 0 | 30 | 6 | 0 |
| 28 | 0 | 0 | 0 | 30 | 6 | 0 |
| 29 | 30 | 6 | 0 | 0 | 0 | 0 |
| 30 | 0 | 0 | 0 | 30 | 6 | 0 |
| 31 | 0 | 0 | 0 | 30 | 6 | 0 |
| 32 | 0 | 0 | 0 | 30 | 6 | 0 |
| 33 | 0 | 0 | 0 | 30 | 6 | 0 |
| 34 | 0 | 0 | 0 | 30 | 6 | 0 |
| 35 | 0 | 0 | 0 | 30 | 6 | 0 |
| 36 | 0 | 0 | 0 | 30 | 6 | 0 |
| 37 | 0 | 0 | 0 | 30 | 6 | 0 |
| 38 | 0 | 0 | 0 | 30 | 6 | 0 |
| 39 | 0 | 0 | 0 | 30 | 6 | 0 |
| 50 | n.a. | n.a. | n.a. | n.a. | n.a. | n.a. |

* - dead mink swab samples tested in pools of 5.

n.a. - no data available.

**Supplementary Table 3.** The number of adult (more than 1-year-old) and juvenile (less than 1-year-old) live minks tested by real-time RT-PCR and found positive at sampled Lithuanian mink farms in November-December 2021.

| **Farm No.** | **No. of tested adult live mink** | **No. of positive adult live mink** | **No. of tested juvenile live mink** | **No. of positive juvenile live mink** |
| --- | --- | --- | --- | --- |
| 6 | n.a. | 0 | n.a. | 0 |
| 8 | 0 | 0 | 30 | 3 |
| 9 | 5 | 0 | 25 | 2 |
| 10 | n.a. | n.a. | n.a. | n.a. |
| 11 | 0 | 0 | 30 | 1 |
| 12 | 6 | 4 | 24 | 9 |
| 13 | 0 | 0 | 30 | 0 |
| 14 | 0 | 0 | 30 | 0 |
| 16 | 30 | 0 | 0 | 0 |
| 18 | n.a. | 0 | n.a. | 0 |
| 19 | n.a. | 0 | n.a. | 0 |
| 26 | 15 | 0 | 15 | 0 |
| 30 | 0 | 0 | 30 | 0 |
| 32 | 4 | 0 | 26 | 0 |
| 33 | n.a. | 0 | n.a. | 0 |
| 36 | 0 | 0 | 30 | 0 |
| 37 | 0 | 0 | 30 | 0 |
| 39 | 0 | 0 | 30 | 0 |
| 40 | 5 | 0 | 25 | 0 |
| 41 | 0 | 0 | 30 | 0 |
| 42 | n.a. | 0 | n.a. | 0 |
| 43 | n.a. | 0 | n.a. | 0 |
| 44 | 0 | 0 | 30 | 0 |
| 45 | 0 | 0 | 30 | 0 |
| 46 | n.a. | 0 | n.a. | 0 |
| 47 | 0 | 0 | 30 | 0 |
| 48 | 11 | 0 | 0 | 0 |
| 49 | n.a. | 0 | n.a. | 0 |
| 51 | 0 | 0 | 30 | 0 |
| 52 | n.a. | 0 | n.a. | 0 |
| 53 | n.a. | 0 | n.a. | 0 |
| 54 | 0 | 0 | 30 | 0 |
| 55 | n.a. | 0 | n.a. | 0 |
| 56 | n.a. | 0 | n.a. | 0 |
| 57 | 0 | 0 | 30 | 0 |

n.a. - no data available.

**Supplementary Table 4.** The number of adult (more than 1-year-old) and juvenile (less than 1-year-old) minks tested by ELISA and found positive at sampled Lithuanian mink farms in November-December 2021.

| **Farm No.** | **No. of tested adult mink** | **No. of positive adult mink** | **No. of tested juvenile mink** | **No. of positive juvenile mink** |
| --- | --- | --- | --- | --- |
| 1 | 0 | 0 | 30 | 4 |
| 2 | 0 | 0 | 30 | 11 |
| 3 | 0 | 0 | 30 | 25 |
| 10 | n.a. | n.a. | n.a. | n.a. |
| 14 | 0 | 0 | 30 | 26 |
| 15 | 0 | 0 | 30 | 19 |
| 16 | 30 | 10 | 0 | 0 |
| 17 | 0 | 0 | 30 | 20 |
| 18 | n.a. | n.a. | n.a. | n.a. |
| 19 | n.a. | n.a. | n.a. | n.a. |
| 20 | n.a. | n.a. | n.a. | n.a. |
| 21 | n.a. | n.a. | n.a. | n.a. |
| 22 | 30 | 19 | 0 | 0 |
| 23 | 0 | 0 | 30 | 2 |
| 24 | n.a. | n.a. | n.a. | n.a. |
| 25 | 0 | 0 | 30 | 29 |
| 26 | 19 | 0 | 11 | 0 |
| 27 | 0 | 0 | 30 | 0 |
| 28 | 0 | 0 | 30 | 0 |

n.a. - blood samples were collected but no information about the age of minks was provided.
